# The Salaries of Deputising Medical Officers

**Published:** 1914-10-24

**Authors:** 


### THE SALARIES OF DEPUTISING MEDICAL OFFICERS.

The above subject is of such special interest just now when the war has caused so many temporary absentees from their posts that it is worthy fuller consideration than could be given in the Note last week, wherein we drew attention to the resolution of the Wandsworth Board of Guardians to award a gratuity of £50 to Dr. Keates for managing the infirmary during the illness of the medical superintendent. One of the advocates of the payment of the gratuity asserted that it was the general practice to pay a deputising officer half the salary of his chief. We doubt if much evidence could be adduced in support of this statement, at least so far as the public medical services are concerned. The only general rule on the subject is that it is not customary to pay any gratuity to a medical officer acting as deputy for his superior when the absence of the latter is for a short period or is part of the conditions of the service. On the other hand, when the absence is prolonged and is owing to exceptional causes, such as illness, the rule has been to pay a gratuity arbitrarily fixed, but usually having some relation to the time of absence and varying from a small sum to the full amount of the salary. In fixing the amount of the gratuity it has been usual to take into account the amount of the assistance which the deputising officer has received; whether he has been required to perform his own duties as well, or a temporary officer has been appointed to carry them out. As regards the special case at Wandsworth we think that the Guardians' proposal was a very reasonable and fair one.

It is the exceptional circumstances arising out of the war and the formulation of principles to guide administrative boards and medical officers that we deal with here. Here, perhaps, we may be permitted to digress for a moment in order to refer to a statement made by one of the opponents to the gratuity at Wandsworth that he knew of some doctors in the Navy who had to be lashed to their places aboard ship. The deduction the speaker evidently desired to be drawn from this was that, whilst doctors on active service were undergoing such hardships, their colleagues in civilian employ should take their share in the burden by renouncing any claim to remuneration which they might have, in equity, for the additional work that they might be called upon to perform. This argument, it is obvious, would apply more to the cases which we are now considering than to the case in which it was used. We think, however, that the argument is an untenable one. Medical men know that active service must entail danger and privation, and in volunteering they set off against them the honour and interest of the work. Civilian employment brings with it no such rewards, and the recompense must be, as it always has been, mainly a monetary one. "Business as usual" obtrudes itself indeed, to sum up admirably the position as regards the medical services.

The staffs are too small to allow one officer to do the work of two for any lengthened period, and unfortunately absence on war service is certain to be long. Most committees have decided to pay officers volunteering for the Navy or Army their full salary, less the Government pay and allowances. This means in the case of nearly all medical officers, except the very small number who have joined the combatant ranks, that they will receive no payment on account of the offices which they have temporarily relinquished. There can, therefore, be no question that the deputy officers appointed should receive the full salary of the vacant posts. No extra cost will be involved except in those few exceptional cases where senior officers have volunteered for or been called to the Colours. Even here the principle, however, should hold good, for, if the deputy be capable of efficiently carrying out the duties, he should receive the salary allotted to the work. In the case of some of the junior posts in the Poor-Law medical service, indeed, it may be impossible to obtain deputies at the old salaries, and higher salaries may have to be given.

